# Treatment of Cardiovascular Dysfunction with PDE3-Inhibitors in Moderate and Severe Hypothermia—Effects on Cellular Elimination of Cyclic Adenosine Monophosphate and Cyclic Guanosine Monophosphate

**DOI:** 10.3389/fphys.2022.923091

**Published:** 2022-07-13

**Authors:** Adrina Kalasho Kuzmiszyn, Anders Lund Selli, Natalia Smaglyukova, Timofei Kondratiev, Ole-Martin Fuskevåg, Roy Andre Lyså, Aina Westrheim Ravna, Torkjel Tveita, Georg Sager, Erik Sveberg Dietrichs

**Affiliations:** ^1^ Norwegian Air Ambulance Foundation, Research and Development Department, Oslo, Norway; ^2^ Experimental and Clinical Pharmacology, Department of Medical Biology, UiT The Arctic University of Norway, Tromsø, Norway; ^3^ Division of Surgical Medicine and Intensive Care, University Hospital of North Norway, Tromsø, Norway; ^4^ Anesthesia and Critical Care Research Group, Department of Clinical Medicine, UiT The Arctic University of Norway, Tromsø, Norway; ^5^ Department of Laboratory Medicine, Division of Diagnostic Services, University Hospital of North Norway, Tromsø, Norway; ^6^ Center for Psychopharmacology, Diakonhjemmet Hospital, Oslo, Norway

**Keywords:** hypothermia, phosphodiesterase 3 inhibitor, phosphodiesterase 3, ATP-binding cassette transporter, cyclic AMP, cyclic GMP, cardiovascular dysfunction

## Abstract

**Introduction:** Rewarming from accidental hypothermia is often complicated by hypothermia-induced cardiovascular dysfunction, which could lead to shock. Current guidelines do not recommend any pharmacological treatment at core temperatures below 30°C, due to lack of knowledge. However, previous in vivo studies have shown promising results when using phosphodiesterase 3 (PDE3) inhibitors, which possess the combined effects of supporting cardiac function and alleviating the peripheral vascular resistance through changes in cyclic nucleotide levels. This study therefore aims to investigate whether PDE3 inhibitors milrinone, amrinone, and levosimendan are able to modulate cyclic nucleotide regulation in hypothermic settings.

**Materials and methods:** The effect of PDE3 inhibitors were studied by using recombinant phosphodiesterase enzymes and inverted erythrocyte membranes at six different temperatures—37°C, 34°C, 32°C, 28°C, 24°C, and 20°C- in order to evaluate the degree of enzymatic degradation, as well as measuring cellular efflux of both cAMP and cGMP. The resulting dose-response curves at every temperature were used to calculate IC_50_ and Ki values.

**Results:** Milrinone IC_50_ and Ki values for cGMP efflux were significantly lower at 24°C (IC_50_: 8.62 ± 2.69 µM) and 20°C (IC_50_: 7.35 ± 3.51 µM), compared to 37°C (IC_50_: 22.84 ± 1.52 µM). There were no significant changes in IC_50_ and Ki values for enzymatic breakdown of cAMP and cGMP.

**Conclusion:** Milrinone, amrinone and levosimendan, were all able to suppress enzymatic degradation and inhibit extrusion of cGMP and cAMP below 30°C. Our results show that these drugs have preserved effect on their target molecules during hypothermia, indicating that they could provide an important treatment option for hypothermia-induced cardiac dysfunction.

## 1 Introduction

Cold-related deaths have been recognized for several hundred years but it was not until late 19th century that hypothermia was defined, when clinical thermometry was made available (Guly, 2011). Today, accidental hypothermia is defined as an unintentional drop in core temperature below 35°C (Brown et al., 2012; Musi et al., 2021). Further, accidental hypothermia is classified in four different stages by The European Resuscitation Council -stage I—*mild hypothermia* (35°C–32°C), stage II—*moderate hypothermia* (32°C–28°C), stage III—*severe hypothermia* (core temperature below 28°C), and stage IV—*severe hypothermia* with no apparent vital signs (core temperature variable) (Lott et al., 2021). Mortality rates approach 40% in severe (<28°C) accidental hypothermia (Vassal et al., 2001; Van der Ploeg et al., 2010). One of the conditions that contribute to the high mortality is hypothermia-induced cardiac dysfunction (HCD) (Han et al., 2010), which is characterized by a decrease in cardiac output, in combination with profound increase in systemic vascular resistance (SVR) when rewarming patients. In worst case scenario, acute cardiovascular failure, so called *rewarming shock,* ensues (Blair, Montgomery and Swan, 1956; Tveita et al., 1996).

Various cardiovascular drugs used to treat heart failure and control blood pressure, target cyclic adenosine monophosphate (cAMP) and cyclic guanosine monophosphate (cGMP). These are intracellular second messengers that are involved in numerous important processes, such as modulating cardiac contraction and vasoregulation. Intracellular levels of these cyclic nucleotides are regulated by the rate of synthesis and degradation by various phosphodiesterases and cellular efflux by transmembrane transporter proteins. Phosphodiesterase 3A (PDE3A), which is especially active in the cardiovascular system, is the main enzyme responsible for degrading cAMP. PDE5A, which resides abundantly in smooth muscle cells in the vascular system, metabolizes primarily cGMP (Omori and Kotera, 2007; Ya et al., 2016). cAMP and cGMP levels are also regulated through active transportation facilitated by transmembrane proteins. These ATP-binding cassette (ABC) transporters are one of the largest transport protein superfamilies (Vasiliou, Vasiliou and Nebert, 2009). ABCC4, which is present in many tissues, transports cAMP out of the cell and has an additional transport site with high Km for both cAMP and cGMP. ABCC5 transports cGMP with high affinity and cAMP with low affinity (Sager and Ravna, 2009).

Well-known β-receptor agonists, such as epinephrine and isoproterenol, increase cAMP levels through the adenylyl cyclase (AC) pathway. Studies in a rat model of severe hypothermia suggest that these drugs increase SVR without simultaneous positive inotropic effect (Dietrichs, Sager and Tveita, 2016) but species-dependent differences exist (Mohyuddin et al., 2021). More promisingly, *in vivo* studies performed in rat models, have demonstrated that PDE3 inhibitors, such as milrinone and levosimendan, have positive effects on stroke volume and cardiac output, almost restoring these parameters to baseline levels during rewarming (Detrichstrichs et al., 2014a; Dettrichstrichs et al., 2014b). Their mechanism of action is through inhibiting cyclic nucleotide breakdown, in contrast to the adrenergic agents, which stimulate their synthesis (Movsesian, 2016). The additional and positive effect of vasodilation by PDE3-inhibitors that was observed in these studies, have prompted research where sodium nitroprusside, a widely known nitric oxide (NO) donor, was studied in the same rat model. The results demonstrate clearly that reducing SVR, without directly supporting the heart, lead to a significant increase in cardiac output (Håheim et al., 2017). These findings indicate that, to treat HCD, it could be favourable to support the failing heart through an alternative route to that of the β-adrenoceptor agonists, and also promote attenuation of SVR. Thus, in the present study we wanted to investigate how PDE3-inhibitors affect cyclic nucleotide regulation in human cells during hypothermia, to improve treatment of severely hypothermic patients.

## 2 Materials and Methods

### 2.1 Temperature

The chosen temperatures of this study were selected to represent the full spectrum of severity, including normothermia; 37–34—32–28—24–20°C. Temperature was maintained at the chosen level by using a Grant Optima T100 heated circulating bath (Grant Instruments LTD., Shepreth, England). Separate experiments were carried out for all included temperatures and parallels. Exposure to the selected temperature lasted for 30 min.

### 2.2 Pharmaceuticals

Amrinone (Sigma-Aldrich, Steinheim, Germany), milrinone (United States Pharmacopeia (USP) Reference Standard, Rockville, United States), and levosimendan (Sigma-Aldrich, Steinheim, Germany) were used in seven different concentrations throughout the study, increasing by a factor 10 and ranging from 1.00E-09 to 1.00E-03 M (1 nM–1 mM), respectively.

### 2.3 Cells

Erythrocytes were chosen for the evaluation of intracellular access of the three drugs as well as cellular extrusion of cyclic nucleotides during hypothermia, since they contain both ABCC4 and ABCC5 transport molecules in the cell membrane (Köck et al., 2007). Blood was provided by Blodbanken (Department of Immunohematology and Transfusion Medicine, University Hospital of North Norway) where all participants were pre-screened and only admitted as donors if they were healthy. The blood was consistently collected from randomly assigned, pre-screened healthy blood donors. As described in our recent publication from the same project (Selli et al., 2021), the regional ethical committee found that ethical review and approval was not required for this study, as the study was performed according to local legislation and institutional requirements included in our agreement with Department of Immunohematology and Transfusion Medicine, University Hospital of North Norway. The participants provided their written informed consent to contribute before sampling at Blodbanken, and we only received anonymized blood samples for the experiments.

### 2.4 Experimental Protocols

Three independent experiments were conducted to determine the degree of intracellular access of the substances and cyclic nucleotide turnover. Each medication and control solution were tested in triplicates and at three independent experiments for each temperature. The study protocol has been described previously by our research group (Selli et al., 2021).

#### 2.4.1 Intracellular Access

Fresh (<24 h) EDTA blood from healthy donors (*n* = 18) was obtained and washed with Krebs-Ringer-Phosphate-Buffer containing glucose (KRPB/G, pH 7.4) and centrifuged (10 min, 1,000 g) three consecutive times. Plasma and buffy coat were removed. KRPB/G was added to the cell solution to bring it to the ratio 1:2.5. Hematocrit (Hct) was measured and adjusted with additional buffer to reach 0.44, in order to give the final Hct of 0.40 in the incubation solution. 500 µl cell suspension (Hct 0.44) were added to test tubes, along with 50 µl of either milrinone (final concentration 10 µM), amrinone (final concentration 100 µM), levosimendan (final concentration 1 µM) or MQ-water (negative control) and incubated for 30 min in the chosen temperature. The reaction was stopped by placing the test tubes on ice and adding 4 ml of ice cold KRFB/G. The samples were subsequently centrifugated (5 min, 600 g), then washed with KRFB/G once more and centrifugation was repeated twice. 50 µl of the remaining red blood cell suspension was transferred to Eppendorf tubes. 50 µl 500 nM IS-Milrinone-d3 was added as an internal standard (TLC Pharmaceutical Standards Ltd.). 200 µl ZnSO_4_ was added to each test sample, to induce lysis of the cells, and mixed in a vortex mixer. 30 µl of the samples were used for measurement of protein concentration, the rest was mixed with 500 µl acetonitrile and centrifugated (2 min, 13400 g). Finally, 100 µl of the solution was collected for analysis using mass spectrometry (MS).

#### 2.4.2 Cellular Efflux

The transport assay was performed using so called inside-out vesicles (IOVs), which were prepared from erythrocytes by using a modified version of the Steck IOV preparation (Steck, 1974). Freshly collected EDTA blood from healthy donors (*n* = 35) was prepared at 0°C–4°C, starting by separating the erythrocytes from plasma by centrifugation at 2,300 g for approximately 15 min. Plasma and buffy were disposed of and the remaining cells washed three times with 5 mM Tris- HCl and 113 mM KCl (pH 8.1), and subsequently centrifugated at 1,000 g. Lysis followed thereafter, with ten volumes of 5 mM Tris-HCl, 0.5 mM EGTA, 4 mM KCl (pH 8.1) and washed by repeated centrifugation at 20,000 g for 20 min and resuspension in the same buffer. Vesiculation was initiated by adding 39 volumes of a hypertonic buffer (0.5 µM Tris-HCl, pH 8.2) to one volume of cell suspension and completed by forcing the solution five times through a 27-gauge syringe needle to promote homogenization of the membranes. The resulting IOVs were separated from the right-side vesicles and ghosts by ultracentrifugation (100,000 g) overnight, using a density gradient, ranging from 1.048 g/ml to 1.146 g/ml (Nycodenz, Axis-Shield PoC, Oslo, Norway) in 5 mM Tris, 3 mM KCl, and 0.3 mM, EGTA. This procedure gathered the IOVs in the uppermost band, which was collected, washed and resuspended in 1.47 mM KH_2_PO_4_, 81 mM K_2_KPO_4_ and 140 mM KCl (pH 7.6). Sidedness was verified by using the acetylcholinesterase accessibility test (Ellman, 1961). The resulting IOVs, which still contained ABC-transporters in the membrane, enabled us to control the intracellular environment, in this case the surrounding medium, and collect the cyclic nucleotides within. Thus, IOVs were subsequently incubated for 60 min at the designated temperature, with or without 2.0 mM ATP, in the following mixture: 20 mM Tris-HCl, 10 mM MgCl_2_, 1 mM EGTA, 121 mM KCl. Radioactive labeled (^3^H)-cAMP or (^3^H)-cGMP (Perkin Elmer, Boston, MA, United States) were added to the incubation solutions in the concentration 20 and 2 μM, respectively, depending on the transporter examined, together with the appointed study drug in concentrations up to 1 mM. The assay was terminated by adding ice cold buffer (<4°C), containing 1.47 mM KH_2_PO_4_, 8.1 mM K_2_HPO_4_, and 140 mM KCl (pH 7.6). Next step was to collect the IOVs, which was done by filtration through a nitrocellulose membrane (Bio-Rad Laboratories, Feldkirchen, Germany), and then drying it. The resulting collection of radioactivity upon the filter, was later quantified by using a Packard TopCount NXT (Packard, Downers Grove, IL, United states) after adding scintillation fluid (MicroScint-O, PerkinElmer, Groningen, Netherlands).

#### 2.4.3 Phosphodiesterase Assay

The study drugs were tested upon their ability to hinder cAMP and cGMP hydrolysis by inhibiting PDE3 and PDE5 respectively. Either 5 µM cAMP or cGMP (Sigma-Aldrich, St. Louis, MO, United States) were used as substrates for the enzymes. The reaction was initiated by adding PDE to the assay solution in designated Eppendorf tubes, containing fresh incubation buffer (10 mM Tris, 8.2 mM Propionic acid, 3 mM Magnesium acetate, 1.5 mM EGTA and 0.5 mg/ml BSA, 0.2 mM DTT) selected substrate (cAMP or cGMP) and inhibitor. The reaction was initiated by adding 0.016 units/µl incubate of PDE3 (Abcam, Cambridge, United Kingdom), or 0.022 units/µl incubate of PDE5 (Sigma-Aldrich, St. Louis, United States). Control samples were free of drug and were either with or without PDE. The incubation time was 30 min in the selected temperature. Reaction was stopped by adding 99.9% methanol to the solutions. Internal standards of cGMP-13C5, cAMP-13C5, AMP-13C5 (Toronto Research Chemicals Inc., Ontario, Canada) and GMP-15N5 (Sigma-Aldrich, St. Louis, MO, United States) were added to each sample, before MS analysis.

### 2.5 Mass Spectrometry Analysis

Liquid chromatography tandem mass spectrometry (LC-MS) was used to assess concentrations of the inhibitors for experiments performed to determine intracellular access of these drugs, as well as to quantify levels of cAMP/AMP and cGMP/GMP for the PDE3-and PDE5-inhibition plots. Internal standards were used, as described previously. There was a linearity from 0.2 nM to at least 2000 nM (r^2^ > 0.998) for cAMP, AMP, and cGMP. For GMP, linearity was present from 2 nM to at least 2000 nM (r^2^ > 0.998). Concerning PDE3-inhibitors, the linearity was found at 10 nM to at least 5,000 nM (r^2^ > 0.99). Lower limit of quantification (LLOQ) was 0.2 for cAMP, cGMP, and AMP, 2 nM for GMP and 10 nM for PDE3-inhibitors (2 µl injection volume).

### 2.6 Bioactivity

IC_50_ and K_i_ values were calculated for each of the drugs, both for their ability to inhibit cAMP- and cGMP- efflux, as well as PDE3 and PDE5 activity. IC_50_ values were derived according to Chou (1976), and K_i_ values according to Cheng and Prusoff (1973). Inhibition curves were created accordingly, both with and without adjustment for normothermic control.

### 2.7 Statistical Analysis

Intracellular concentrations of drugs were adjusted for protein concentrations in each sample. The total incubation concentrations were also adjusted for protein concentrations to evaluate the degree of access in percentage. Statistical analysis was performed using one-way ANOVA with Holm-Sidak multiple comparisons post hoc test at each temperature. ANOVA on ranks with Dunn post hoc test was used when the results were not normally distributed. The results are presented as means ± standard error of mean (SEM). *p*-values < 0.05 were considered significant results. Degrees of freedom (dF) is given as between group values. SigmaPlot 14.0 (Systat Software, San Jose, CA, United States) was used for all analysis, as well as creating the graphs.

## 3 Results

### 3.1 Intracellular Access

**FIGURE 1:**
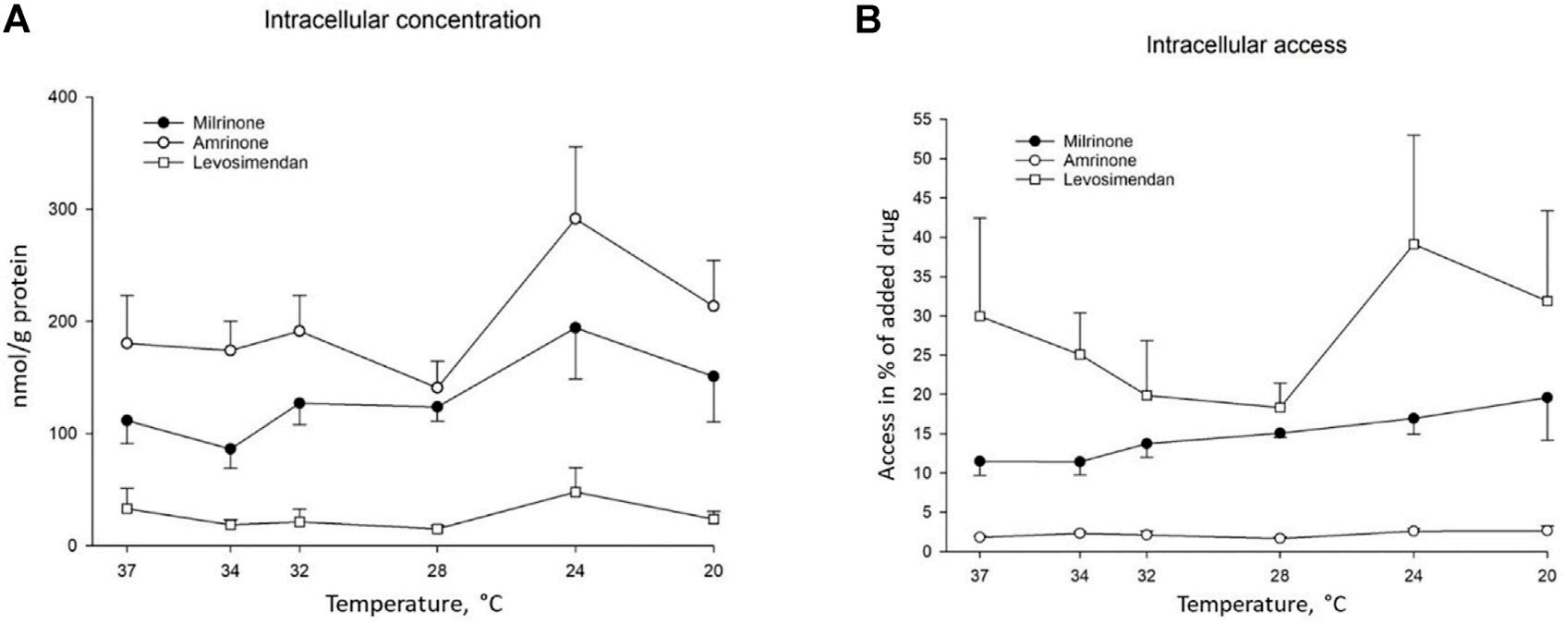
Temperature-dependent intracellular access of added milrinone, amrinone, and levosimendan, presented as means ± SEM **(A)** Intracellular concentration of milrinone, amrinone, and levosimendan in nmol/g protein at temperatures ranging from 37°C to 20°C, *n* = 3. **(B)** Intracellular access of milrinone, amrinone, and levosimendan in % of drug concentration per gram protein, compared to drug concentration per gram protein in the incubation solution at temperatures ranging from 37°C to 20°C, *n* = 3.

After 30 min of incubation, all study drugs were found in the IOVs at all temperatures. Decreasing the temperature from 37°C to 20°C did not significantly change their ability to reach their intracellular site of action (Figure 1). A higher percentage of levosimendan entered the cells compared to milrinone and amrinone at 34°C (25.06 ± 5.289 vs. 11.41 ± 1.675, ANOVA, dF = 2, F = 12.755, *p* = 0.047, and 2.303 ± 0.284, ANOVA, dF = 2, F = 12.755, *p* = 0.007), as well as at 28°C compared to amrinone (18.29 ± 3.122 vs. 1.66 ± 0.216, ANOVA, dF = 2, F = 23.133, *p* = 0.002). The degree of intracellular access was also significantly higher for milrinone, compared to amrinone, at 28°C (15.06 ± 0.544 vs. 1.66 ± 0.216, ANOVA, dF = 2, F = 23.133, *p* = 0.004).

### 3.2 Intracellular Elimination by Phosphodiesterase

**FIGURE 2:**
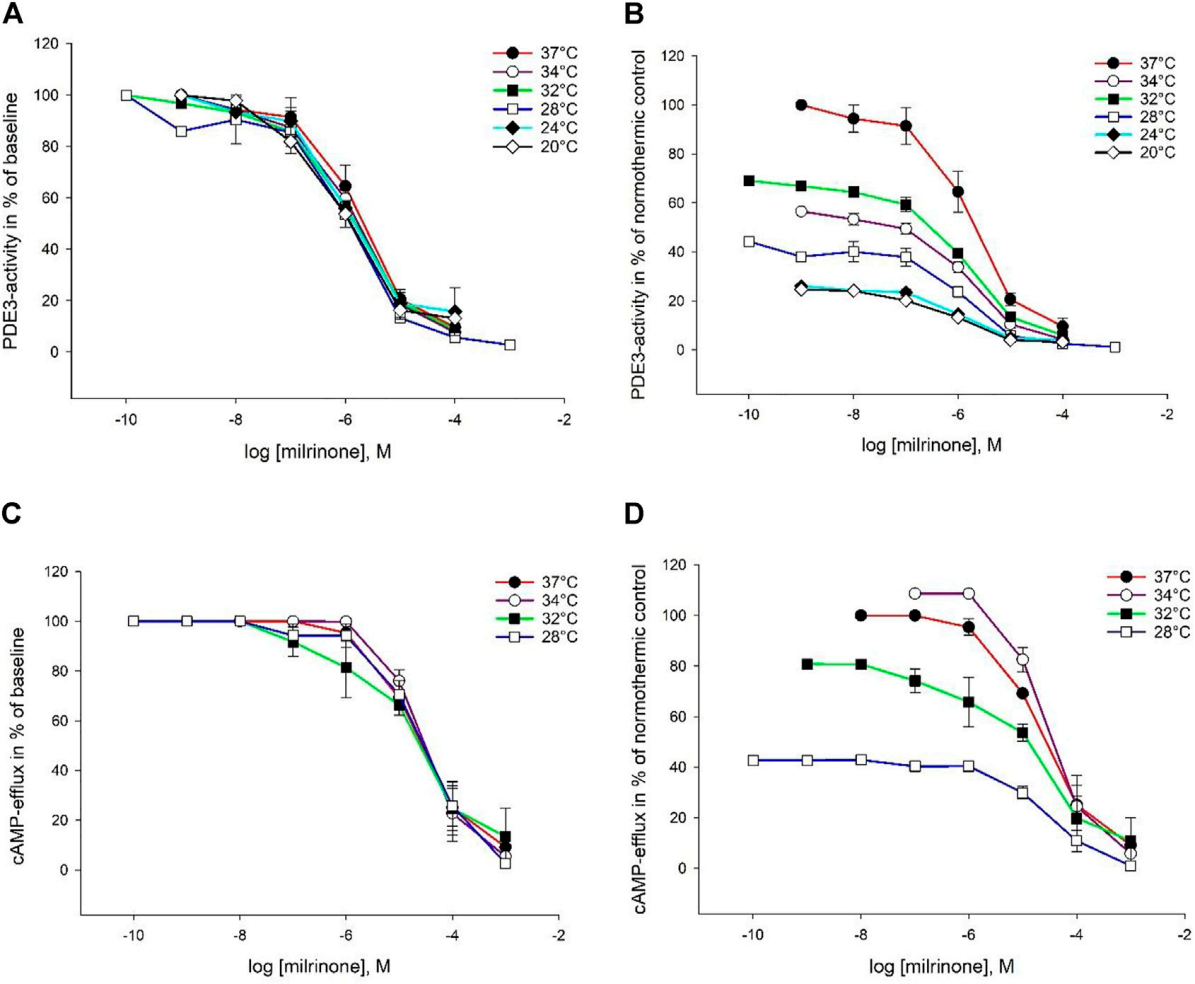
Temperature-dependent inhibition of PDE3 and cAMP-efflux by milrinone. The doses are in logarithm of the concentration in mol/L, presented as means ± SEM. **(A)** Inhibition curves for PDE3-activity at temperatures ranging from 37°C to 20°C. **(B)** Inhibition curves for PDE3-activity in % of normothermic inhibition curve at temperatures ranging from 37°C to 20°C. **(C)** Inhibition curves for ABCC4-activity at temperatures ranging from 37°C to 20°C. **(D)** Inhibition curves for ABCC4-activity in % of normothermic inhibition curve at temperatures ranging from 37°C to 20°C.

**FIGURE 3:**
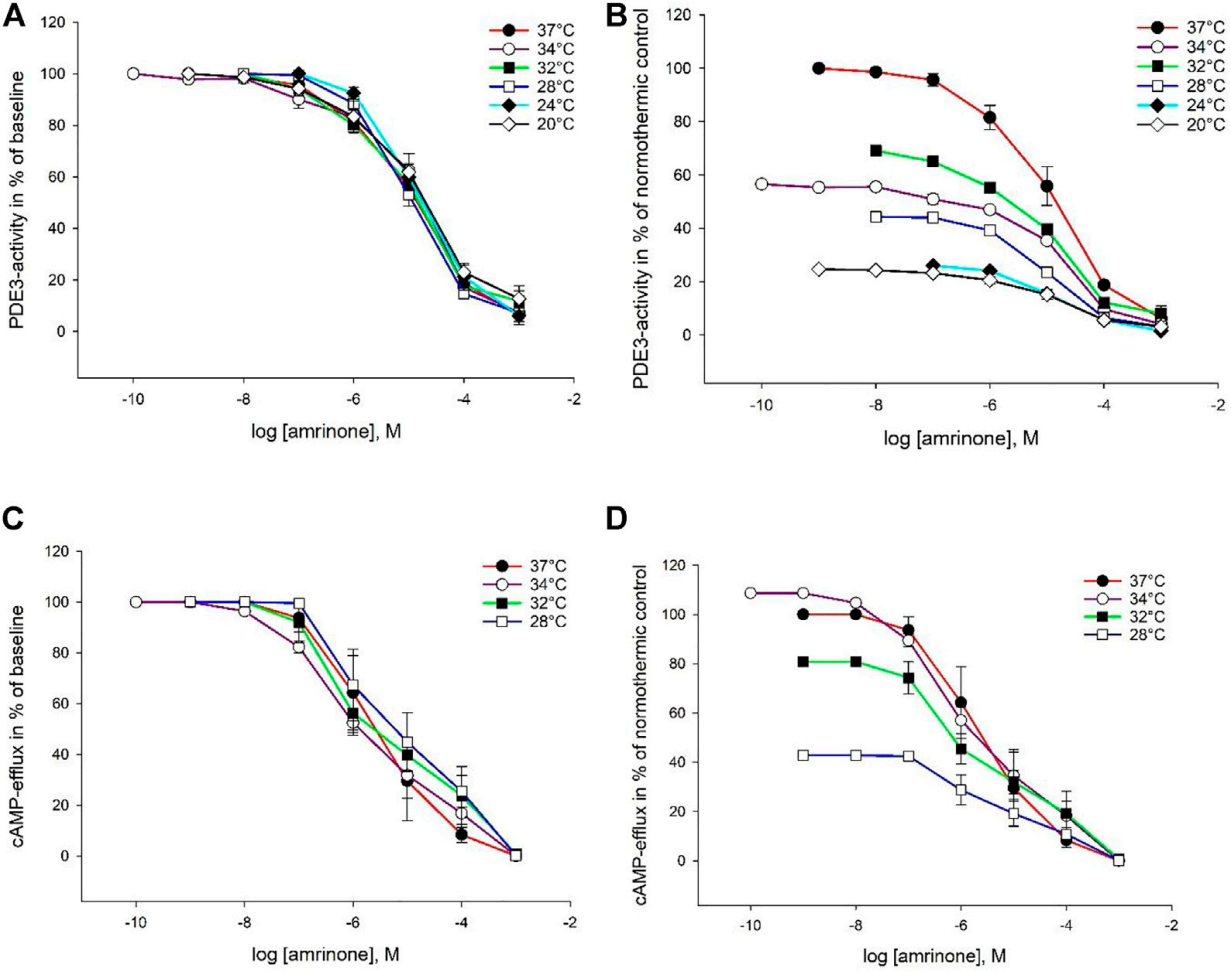
Temperature-dependent inhibition of PDE3 and cAMP-efflux by amrinone. The doses are in logarithm of the concentration in mol/L, presented as means ± SEM. **(A)** Inhibition curves for PDE3-activity at temperatures ranging from 37°C to 20°C. **(B)** Inhibition curves for PDE3-activity in % of normothermic inhibition curve at temperatures ranging from 37°C to 20°C. **(C)** Inhibition curves for ABCC4-activity at temperatures ranging from 37°C to 20°C. **(D)** Inhibition curves for ABCC4-activity in % of normothermic inhibition curve at temperatures ranging from 37°C to 20°C.

**FIGURE 4:**
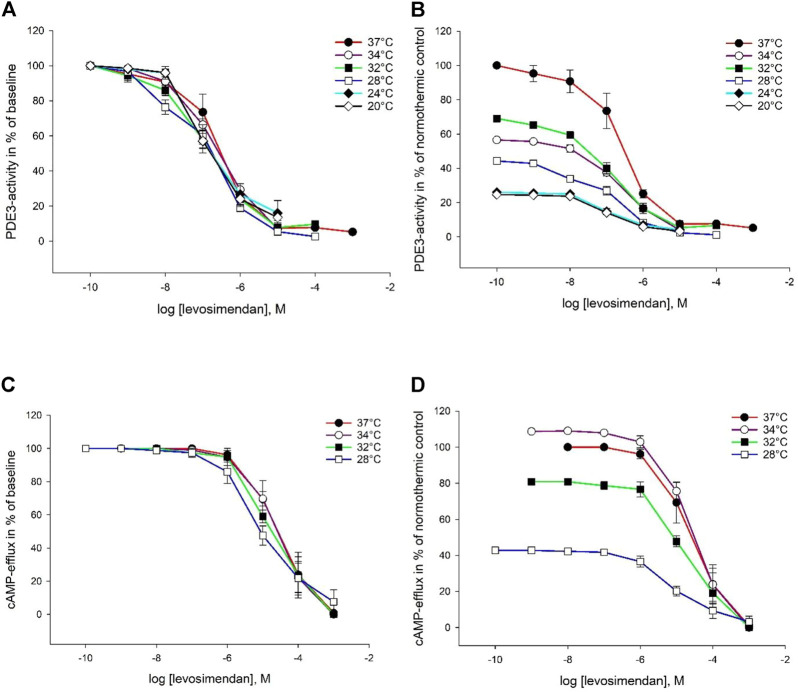
Temperature-dependent inhibition of PDE3 and cAMP-efflux by levosimendan. The does are in logarithm of the concentration in mol/L, presented as means ± SEM. **(A)** Inhibition curves for PDE3-activity at temperatures ranging from 37°C to 20°C. **(B)** Inhibition curves for PDE3-activity in % of normothermic inhibition curve at temperatures ranging from 37°C to 20°C. **(C)** Inhibition curves for ABCC4-activity at temperatures ranging from 37°C to 20°C. **(D)** Inhibition curves for ABCC4-activity in % of normothermic inhibition curve at temperatures ranging from 37°C to 20°C.

Milrinone, amrinone, and levosimendan were all able to inhibit both PDE enzymes at all six temperatures. Inhibition plots for PDE3 are depicted in Figures 2–4. IC_50_ and K_i_ values for PDE3 inhibition were not significantly different at hypothermia and the same was true for PDE5 inhibition. IC_50_ and K_i_ values for PDE3 inhibition by amrinone were significantly higher, regardless of temperature, compared to levosimendan (Table 2). At 34°C and 32°C, there were significant differences in values between amrinone and milrinone (IC_50_: 9.863 ± 1.709 µM vs. 1.771 ± 0.716 µM, ANOVA, dF = 2, F = 23.056, *p* = 0.004 and 15.07 ± 1.855 µM vs. 1.302 ± 0.357 µM, ANOVA, dF = 2, F = 56.783, *p* < 0.001). For PDE5 inhibition, milrinone had consistently higher IC_50_ and K_i_ values, compared to amrinone (Table 1), and they were also significantly higher at 24°C and 20°C, compared to levosimendan (IC_50_: 253 ± 32.92 µM vs. 148 ± 26.12 µM, ANOVA, dF = 2, F = 10.394, *p* = 0.045 and 330 ± 58.11 µM vs. 157 ± 27.45 µM, ANOVA, dF = 2, F = 9.906, *p* = 0.034).

**TABLE 1:** IC_50_ and K_i_ values for inhibition of PDE3, PDE5, ABCC4, and ABCC5 at temperatures ranging from 37°C to 20°C.

Milrinone								
	PDE3		PDE5		ABCC4		ABCC5	
Temperature (°C)	IC_50_	K_i_	IC_50_	K_i_	IC_50_	K_i_	IC_50_	K_i_
37	2.99 ± 1.28	0.14 ± 0.059	336 ± 58.60^‡^	85.3 ± 14.87^‡^	58.9 ± 13.09^‡●^	35.69 ± 7.94 ^‡●^	22.8 ± 1.52	12.91 ± 0.86
34	1.77 ± 0.72 ‡	0.081 ± 0.033^‡^	255 ± 29.84^‡^	64.6 ± 7.57^‡^	67.0 ± 22.65^‡^	40.65 ± 13.73^‡^	17,8E ± 1.73^‡^	10.04 ± 0.96^‡^
32	1.30 ± 0.36^‡^	0.060 ± 0.016^‡^	280 ± 48.33^‡^	70.9 ± 12.26^‡^	32.9 ± 14.61	19.95 ± 8.86	17.2 ± 3.02^‡^	9.70 ± 1.71^‡^
28	0.82 ± 0.31	0.038 ± 0.014	305 ± 72.47^‡^	77.4 ± 18.39^‡^	37.4 ± 24.52	22.67 ± 14.86	18.2 ± 2.03^‡●^	10.26 ± 1.15^‡●^
24	1.91 ± 0.73	0.087 ± 0.034	253 ± 32.92^‡●^	64.2 ± 8.35^‡●^	-		8.62 ± 2.69^#^	4.87 ± 1.52^#^
20	2.15 ± 0.65	0.099 ± 0.030	330 ± 58.11^‡●^	83.8 ± 14.75^‡●^	-		7.35 ± 3.51^#^	4.16 ± 1.98^#^

^#^Significant difference (*p*-value < 0.05) when compared to normothermic control.

^‡^Significant difference (*p* < 0.05) when compared to amrinone.

^●^Significant difference (*p* < 0.05) when compared to levosimendan.

Values are presented as means ± SEM and given in µM.

### 3.3 Cellular Efflux

The IC_50_ and K_i_ values for inhibition of cyclic nucleotide extrusion showed no significant differences between 37°C and 28°C for all PDE3 inhibitors. At 24°C and 20°C, however, neither of the study drugs were able to inhibit cAMP-efflux. Milrinone had significantly higher capacity to inhibit cGMP-efflux at 24°C and 20°C, compared to normothermia (IC_50_: 22.8 ± 1.52 µM compared to 8.62 ± 2.69 µM, ANOVA, dF = 5, F = 5.762, *p* = 0.025 and 7.35 ± 3.51 µM, ANOVA, dF = 5, F = 5.762, *p* = 0.014) (Table 1). Amrinone had significantly lower IC_50_ and K_i_ values at 37°C–34°C for cAMP expulsion and at 34°C–28°C for cGMP-efflux, compared to milrinone (Table 2). Levosimendan had significantly higher values at 34°C and 28°C, compared to amrinone, for cGMP-extrusion (Table 3).

**TABLE 2:** IC_50_ and K_i_ values for inhibition of PDE3, PDE5, ABCC4 and ABCC5 at temperatures ranging from 37°C to 20°C.

Amrinone								
	PDE3		PDE5		ABCC4		ABCC5	
Temperature	IC_50_	K_i_	IC_50_	K_i_	IC_50_	K_i_	IC_50_	K_i_
37°C	10.55 ± 3.53^●^	0.48 ± 0.16^●^	110 ± 12.80	27.8 ± 3.25	4.65 ± 3.21*	2.82 ± 1,946*	2.20 ± 0.70	1.24 ± 0.39
34°C	9.86 ± 1.71^●^*	0.45 ± 0.078^●^*	91.9 ± 3.65	23.3 ± 0.93	1.21 ± 0.10*	0.73 ± 0.058*	2.43 ± 0.75^●^*	1.37 ± 0.43^●^*
32°C	15.07 ± 1.86^●^*	0.69 ± 0.085^●^*	84.5 ± 5.94	21.5 ± 1.51	5.49 ± 2.25	3.33 ± 1,362	1.75 ± 0.61*	0.99 ± 0.35*
28°C	15.68 ± 2.25^●^	0.72 ± 0.103^●^	95.0 ± 7.29	24.1 ± 1.85	8.42 ± 4.81	5.11 ± 2.91	1.10 ± 0.33^●^*	0.62 ± 0.18^●^*
24°C	24.87 ± 5.05^●^	1.14 ± 0.23^●^	99.1 ± 4.20	25.1 ± 1.07	-		1.10 ± 0.51	0.62 ± 0.29
20°C	26.44 ± 15.73^●^	1.21 ± 0.72^●^	105 ± 8.04	26.7 ± 2.04	-		5.44 ± 2.68	3.08 ± 1.52

#Significant difference (*p*-value < 0.05) when compared to normothermic control.

*Significant difference (*p* < 0.05) when compared to milrinone.

•Significant difference (*p* < 0.05) when compared to levosimendan.

Values are presented as means ± SEM and given in µM.

**TABLE 3:** IC_50_ and K_i_ values for inhibition of PDE3, PDE5, ABCC4, and ABCC5 at temperatures ranging from 37°C to 20°C.

Levosimendan								
	PDE3		PDE5		ABCC5		ABCC5	
Temperature	IC_50_	K_i_	IC_50_	K_i_	IC_50_	K_i_	IC_50_	K_i_
37°C	0.33 ± 0.10^‡^	0.015 ± 0.00^‡^	155 ± 4.74	39.2 ± 1.20	9.41 ± 5.91*	5.71 ± 3.58*	28.5 ± 10.29	16.1 ± 5.82
34°C	0.29 ± 0.067^‡^	0.013 ± 0.00^‡^	150 ± 13.45	38.0 ± 3.41	12.7 ± 6.60	7.73 ± 4.00	13.9 ± 1.72^‡^	7.86 ± 0.97^‡^
32°C	0.19 ± 0.060^‡^	0 0088 ± 0.00^‡^	162 ± 22.51	41.2 ± 5.71	16.5 ± 5.56	10.0 ± 3.37	13.2 ± 3.99	7.45 ± 2.25
28°C	0.101 ± 0.026^‡^	0.0046 ± 0.00^‡^	183 ± 23.04	46.4 ± 5.85	27.2 ± 21.34	16.5 ± 12.94	11.9 ± 1.35^‡^*	6.74 ± 0.76^‡^*
24°C	0.35 ± 0.21^‡^	0.016 ± 0.01^‡^	148 ± 26.12*	37.6 ± 6.63*	-		18.4 ± 12.73	10.4 ± 7.20
20°C	0.35 ± 0.200^‡^	0.016 ± 0.01^‡^	157 ± 27.45*	39.9 ± 6.97*	-		7.15 ± 1.57	4.04 ± 0.89

^#^Significant difference (*p*-value < 0.05) when compared to normothermic control.

*Significant difference (*p* < 0.05) when compared to milrinone.

^‡^Significant difference (*p* < 0.05) when compared to amrinone.

Values are presented as means ± SEM and given in µM.

### 3.4 Drug Selectivity

#### 3.4.1 Phosphodiesterase Enzymes

Calculated IC_50_ ratios for the phosphodiesterase enzymes showed that notably higher concentrations of all three drugs were needed to inhibit PDE5, compared to PDE3, during normothermia. The same trend was seen at all studied temperatures and appeared to be accentuated for levosimendan from 32°C to 28°C where the IC_50_ ratio for PDE5/PDE3 inhibition doubled from 846 to 1820. Amrinone was the least selective drug with ratios spanning from 10.38 (37°C) to 3.98 (20°C) (Table 4).

**TABLE 4:** Drug selectivity for milrinone (M), amrinone (A), and levosimendan (L) at temperatures ranging from 37°C to 20°C. Values are ratios between IC_50_-values for different elimination ways of cAMP and cGMP. Neither of the drugs inhibited ABCC4-activity below 28°C.

Drug selectivity (IC_50_)/(IC_50_)	(PDE5)/(PDE3) inhibition			(cGMP-efflux)/(cAMP-efflux) inhibition			(cAMP-efflux)/(PDE3) inhibition			(cGMP-efflux)/(PDE5) inhibition		
Temperature	M	A	L	M	A	L	M	A	L	M	A	L
37°C	112	10.38	472	0.39	0.47	3.03	19.66	0.441	28.76	0.068	0.020	0.184
34°C	144	9.32	510	0.27	2.01	1.09	37.86	0.123	43.45	0.070	0.026	0.093
32°C	215	5.61	846	0.52	0.32	0.80	25.27	0.364	85.98	0.061	0.021	0.081
28°C	370	6.06	1820	0.49	0.13	0.44	45.40	0.537	270.7	0.060	0.012	0.065
24°C	133	3.98	427	-	-	-	-	-	-	0.034	0.011	0.124
20°C	154	3.98	450	-	-	-	-	-	-	0.022	0.052	0.045

#### 3.4.2 Cyclic Nucleotide Efflux

Concerning drug selectivity for cGMP/cAMP efflux, milrinone and amrinone appeared to inhibit cGMP extrusion at lower concentrations during normothermic conditions (ratio of 0.39 and 0.47, respectively). Inhibition ratio for levosimendan (3.03) indicated an opposite tendency, but when the temperature decreased, the ratio switched to 0.44 at 28°C.

#### 3.4.3 Cyclic Adenosine Monophosphate Elimination

Higher concentrations (IC_50_) were needed to inhibit ABCC4 compared to PDE3, for milrinone (ratio of 19.66) and levosimendan (28.76) at 37°C. cAMP efflux/PDE3 ratio almost doubled at 34°C for milrinone (37.86) and increased steadily for levosimendan as the temperature fell (270.65 at 28°C). Higher IC_50_ values were consistently registered for inhibiting PDE3-activity by amrinone, compared to ABCC4-function, reflected in an IC_50_-ratio <1 at all temperatures.

#### 3.4.4 Cyclic Guanosine Monophosphate Elimination

The calculated IC_50_ ratios indicated higher selectivity towards cGMP efflux inhibition than PDE5-mediated cGMP elimination for all medications at all temperatures.

## 4 Discussion

The present study shows that both levosimendan, milrinone and amrinone are able to reduce cAMP elimination during cooling to severe hypothermia (20°C). All drugs had intact, inhibitory effect on PDE3 and we observed no pharmacodynamic shift towards more potent effect on cGMP-metabolism relative to cAMP metabolism during hypothermic conditions. Our findings therefore suggest that PDE3-inhibition is a promising target to treat and prevent HCD, already from temperatures down to 20°C in severely hypothermic patients.

Current European guidelines for treating accidental hypothermia, do not recommend use of vasoactive drugs in patients with core temperatures below 30°C. As a result, hypothermic cardiac arrest patients should be treated according to the standard advanced life support (ALS) algorithm, but without any pharmacological aid. Once the core temperature reaches >30°C, it is recommended that adrenaline is administered every 6–10 min (instead of 3–5 min, as in normothermic conditions). When the patient is no longer hypothermic, ALS algorithm is followed as originally intended (Lott et al., 2021). In prehospital settings, the focus is to extricate and impede further cooling. Patients with signs of cardiovascular instability, cardiac arrest, or core temperatures <30°C, are transported directly to a hospital with stand-by extracorporeal life support (ECLS) for active rewarming. Remaining patients are triaged to the nearest, appropriate hospital for passive and/or minimally invasive active rewarming (Lott et al., 2021; Paal et al., 2022).

In accidental hypothermic patients, one pharmacological strategy to prevent or treat HCD is to elevate cardiac contractility (Dietrichs, Sager and Tveita, 2016). Amrinone, a PDE3-inhibitor, that is, also an important precursor for so-called novel cardiotonic agents, providing positive inotropic effect, has been used intravenously in short-term management of cardiac heart failure (Endoh, 2013). The same pharmacological mechanisms are displayed by milrinone, although it is 30 times more potent than amrinone and it is commonly used in intensive care units as a mean to treat cardiogenic shock (Young and Ward, 1988; Mathew et al., 2021). Levosimendan acts as a PDE3 inhibitor at higher concentrations (>0.3 µM). It is also being used for managing acutely decompensated congestive heart failure (Pathak et al., 2013). Being well-known and widely distributed, these three medications can provide means to treat hemodynamically unstable patients in prehospital settings, especially if the transfer time to nearest ECLS centre is several hours. Nations worldwide without ECLS-facilities are inherently at a disadvantage but can make use of treatment protocols with minimally invasive and cheap pharmacological therapy. Additionally, our findings could also improve more advanced ECLS-treatment of hypothermic patients. Human erythrocytes contain both β_1_-and β2-adrenergic receptors, with the latter being predominant (Sager, 1982; Bree et al., 1984). It has been shown that β_2_-receptor activation increases the level of cAMP intracellularly, and that there is a concentration-dependent increase in red blood cell filterability when stimulated with adrenaline (Tuvia et al., 1999; Horga et al., 2000). Thus, decreased elimination of cAMP through ABCC4, could provide a pharmacological strategy to increase erythrocyte-deformability and improve the microcirculation during ECLS-treatment of hypothermic patients.

The evidence for using pharmacological support in cold patients is scarce and the use of, e.g., adrenaline as inotropic support is mainly based on animal studies. The same goes for our study drugs. Various experiments have been conducted, both *in vitro* and *in vivo*. In rodent models, milrinone have a preserved effect on both systolic and diastolic heart function during cooling, down to 15°C and rewarming to 37°C (Tveita and Sieck, 2012; Dietrichs et al., 2014a). On the other hand, in isolated guinea pig hearts, milrinone was shown to have no inotropic effect at study temperatures 31°C and 34°C (Rieg et al., 2009). Interestingly, the latter study demonstrated a temperature-dependent elevation of cardiac inotropy, which could impede any further effect of pharmacological treatment. Studies exploring temperature-dependent effects of levosimendan, show that it has significant positive effects on the circulation in deeply hypothermic rats connected to cardiopulmonary bypass, when compared to adrenaline (Rungatscher et al., 2012). Levosimendan also increases organ blood flow during rewarming, besides contributing to higher CO (Håheim et al., 2020). Knowledge of the effects of amrinone during hypothermia are limited. One study examined whether amrinone could accelerate rewarming in patients after iatrogenic mild hypothermia during neurosurgical procedures (Inoue et al., 2002) but, as of today, no *in vitro* or *in vivo* experiments that investigates potential use of amrinone in accidental hypothermia, exists to our knowledge.

Our findings demonstrate that PDE3-inhibitors milrinone, amrinone, and levosimendan are all able to enter the cells and inhibit PDE3 down to 20°C. Furthermore, we show that their pharmacodynamic effects, in regulating cAMP- and cGMP-elimination, is intact during severe hypothermia and that the dose-response relationship is maintained. It is however likely that the pharmacokinetic properties of these drugs will differ from normothermic temperatures. In general, there is a higher risk of drug and metabolite accumulation in plasma because of diminished clearance at low core temperatures. The volume of distribution is often reduced. This mandates a lower dosage but at the same time, one has to take into account that receptor sensitivity could differ from normothermia (Mallet, 2002; van den Broek et al., 2010). The present study also shows reduced activity of both PDE-enzymes and ABCC-transporters with temperature reduction. Still, all PDE3-inhibitors had intact effect on these target molecules during severe hypothermia. This could provide a more physiological strategy to elevate cAMP levels in hypothermia and avoid an extensive and harmful increase of cAMP, seen with adrenaline-infusion (Dietrichs et al., 2015). Hence, use of PDE3-inhibitors is a promising treatment strategy as their pharmacodynamic effects appear unchanged during hypothermia. Before these drugs are considered for inclusion in guidelines for rewarming accidental hypothermia patients, it is imperative that their temperature-dependent pharmacokinetic properties are established and that their electrophysiological properties are tested in cardiomyocytes. Further, translational studies to assess optimal dosage regimes at different body temperatures and rewarming rates, are therefore necessary to avoid drug toxicity and therapy failure.

## 5 Conclusion

Milrinone, amrinone, and levosimendan are able to cross cell membranes and reach their cytosolic site of action, during severe hypothermia. At temperatures down to 20°C, they have maintained inhibitory effects on inhibiting cellular elimination of cAMP and cGMP, and provide a promising treatment strategy to treat and prevent hypothermia-induced cardiac dysfunction.

## Data Availability

The original contributions presented in the study are included in the article/supplementary material, further inquiries can be directed to the corresponding author.
